# How Many Kinds of Birds Are There and Why Does It Matter?

**DOI:** 10.1371/journal.pone.0166307

**Published:** 2016-11-23

**Authors:** George F. Barrowclough, Joel Cracraft, John Klicka, Robert M. Zink

**Affiliations:** 1 Department of Ornithology, American Museum of Natural History, New York, New York United States of America; 2 Department of Biology and Burke Museum of Natural History and Culture, University of Washington, Seattle, Washington, United States of America; 3 School of Natural Resources, Nebraska State Museum, and School of Biological Sciences, University of Nebraska, Lincoln, Nebraska, United States of America; Consejo Superior de Investigaciones Cientificas, SPAIN

## Abstract

Estimates of global species diversity have varied widely, primarily based on variation in the numbers derived from different inventory methods of arthropods and other small invertebrates. Within vertebrates, current diversity metrics for fishes, amphibians, and reptiles are known to be poor estimators, whereas those for birds and mammals are often assumed to be relatively well established. We show that avian evolutionary diversity is significantly underestimated due to a taxonomic tradition not found in most other taxonomic groups. Using a sample of 200 species taken from a list of 9159 biological species determined primarily by morphological criteria, we applied a diagnostic, evolutionary species concept to a morphological and distributional data set that resulted in an estimate of 18,043 species of birds worldwide, with a 95% confidence interval of 15,845 to 20,470. In a second, independent analysis, we examined intraspecific genetic data from 437 traditional avian species, finding an average of 2.4 evolutionary units per species, which can be considered proxies for phylogenetic species. Comparing recent lists of species to that used in this study (based primarily on morphology) revealed that taxonomic changes in the past 25 years have led to an increase of only 9%, well below what our results predict. Therefore, our molecular and morphological results suggest that the current taxonomy of birds understimates avian species diversity by at least a factor of two. We suggest that a revised taxonomy that better captures avian species diversity will enhance the quantification and analysis of global patterns of diversity and distribution, as well as provide a more appropriate framework for understanding the evolutionary history of birds.

## Introduction

There can scarcely be a datum more critical for biologists to know, or of more intrinsic interest to the public, than the number of species of organisms that surround us [1]. As many authors have noted, identifying the elements of biodiversity is a prerequisite to their conservation. Beginning with early natural historians, an understanding of species diversity was organized and conceptualized. This understanding, codified into the Linnaean taxonomic scheme, formed the basis for scientific studies of speciation, phylogeny, and biogeography, and later came to play a role in species conservation. Over the last two and a half centuries since publication of Linnaeus’ *Systema Naturae*, estimates of the number of species have evolved because of changing philosophies regarding species concepts and, more recently, due to new molecular techniques that better allow discovery of discrete evolutionary units at relatively fine geographic scales [2]. Nevertheless, how much taxonomic diversity is present on Earth remains as one of the most vexing and important of scientific questions [3,4].

A profound confounding factor in estimating species diversity was noted by Mora et al. [5]: “In spite of 250 years of taxonomic classification and over 1.2 million species already catalogued in a central database, our results suggest that some 86% of existing species on Earth and 91% of species in the ocean still await description.” However, this is thought to be less true of higher vertebrates and, for example, birds present a group that is considered to be taxonomically well known, with estimates that more than 95% of their global species diversity has been described. But is this correct? We contend that actual avian evolutionary diversity is less well known than traditionally thought and is, indeed, substantially underestimated. Here, we evaluate differing concepts of species and the relatively recent application of molecular methods in establishing the diversity of the world’s birds. We compare molecular and morphological estimates of species numbers based on two concepts with differing conceptual bases, the biological species concept, which emphasizes the process and consequences of mate choice, and the diagnostic phylogenetic species concept, which focuses on the pattern of character differences to individuate taxa with separate evolutionary histories. We suggest that the latter (i.e. a taxonomy that documents evolutionary history and divergence accurately) best meets the requirements of studies of comparative biology, including studies of speciation and biogeography (enumerating patterns of diversity and endemism), as well as for the ecological and conservation sciences.

## Material and Methods

### Morphological estimates of diversity

Estimates of the number of extant species of birds varied greatly during the first half of the 20th century [6], but stabilized with the check-list begun by Peters [7], edited by Peters, Mayr, Paynter and others through 16 volumes, and completed in 1987 [8]. These volumes treated many taxa as subspecies that had formerly been ranked as species, reflecting the philosophy of the proponents of the polytypic biological species concept prevalent at the time [6]. The completion of the series led to reference lists of the birds of the world [9] that were used in managing museum collections and, with a few updates, a list including 9159 species was adopted by the American Ornithologists' Union (AOU) Committee on Collections [10].

At about this time, molecular surveys of geographic variation began to appear and provide novel data on gene flow and genetic divergence. As a consequence, later compendia, for example, Sibley and Monroe [11], the 16 volumes of del Hoyo et al. [12], and most recently the Howard and Moore checklist[13,14], represent an eclectic mixture [15,16] of traditional morphological taxonomy combined with various molecular assessments of genetic differentiation [16]. These latter studies are still far from complete and, as a result, there currently exists no homogeneous appraisal of avian species taxa in the post-phenotypic era [17].

Using a uniform random number generator, we produced a sample of 200 species from the worldwide list of Wood & Schnell [10] of 9159 biological species of birds (S1 Table). This list reflects species limits as deduced mostly through morphological assessments. We deemed this list appropriate for testing morphological species limits. Although a small number of new species has been discovered since 1986, our purpose was to obtain a random sample such that the future number of newly described species would not measurably influence the study design (see below). In estimating the number of phylogenetic species (see S1 Appendix) within each biological species, we examined all specimens present for each species in the large specimen holdings of the American Museum of Natural History (New York), and in some instances, the collections of the Museum of Natural Science, Louisiana State University (Baton Rouge), and the Field Museum of Natural History (Chicago). Series of specimens representing subspecies or different geographic areas were examined for diagnosable differences (although specific catalog numbers were not recorded, catalog numbers of specimens examined can be obtained from the respective institutions for the species studied). The primary literature on geographic variation, as well as major faunistic works treating regions or relevant taxa, were also consulted. We used qualitative differences in plumage pattern, color, and morphology, including size and shape, as evidence of diagnosably distinct populations (i.e., phylogenetic species), whereas we ignored quantitative differences of size and shade of color. Biological species composed entirely of individuals without discernable qualitative differences within sex or age classes were considered a single phylogenetic species. Species with two or more distinctive morphologies within or among age or sex classes in the same localities were also considered to represent single polymorphic species. Biological species were hypothesized to contain multiple phylogenetic species if, within a single stratum of age or sex, populations showed at least one qualitative character difference that was either geographically disjunct (allopatric) or was continuous only by virtue of a narrow area of character change (gradient or cline) compared to the total geographic ranges of the different forms.

Three authors (GFB, JC, RMZ) individually investigated one third of the species in the random sample, and estimated the number of phylogenetic species included in each of the biological species. We computed the distribution of phylogenetic taxa per biological species for each of the three authors as well as the overall distribution. We used a bootstrap procedure to obtain confidence intervals for the average; this was done by resampling the distribution with replacement 1000 times. We also compared the distributions of the three authors using two sample Kolmogorov-Smirnov tests. To compare the results with generally accepted subspecific taxonomy of birds, we correlated our estimates of the numbers of phylogenetic species with the numbers of described subspecies in the same biological species-level taxon using a standard reference [8].

To estimate the effect of newly discovered species, recent molecular data, and changing taxonomic standards on our estimate of the number of phylogenetic species, we examined the classification of our sample of 200 species in the recent Howard and Moore checklists [13,14]. These lists comprise 10021 species that are partly based on phenotypic and partly based on genetic results. We determined whether each of the 200 species was treated identically to the AOU list, was lumped, split, or was otherwise altered in taxonomic treatment. In this way we obtained a ratio of phylogenetic species per biological species for the Howard and Moore 2013 and 2014 estimates of 10021 taxa.

### Genetic estimates of diversity

For a more focused assessment of the effect of molecular studies on estimates of avian diversity, we surveyed the literature on phylogeographic studies, looking for variation and differentiation in rapidly evolving mitochondrial DNA (mtDNA) genes for species that had been well sampled from throughout their geographic range. In all we identified 437 biological species that were included in such studies and summarized the number of reciprocally monophyletic, geographically contiguous groupings within each (S2 Table). For analytical purposes we assume that these reciprocally monophyletic populations or groups of populations provided a proxy for phylogenetic species. This meta-analysis provides an independent genetic assessment of how many independent evolutionary lineages exist per biological species [18–20], and complements the morphological study.

We note two caveats. Species are generally not sampled randomly for molecular assessment of geographic structure (phylogeography). In general, investigators tend to choose species that they suspect *a priori* exhibit interesting patterns of geographic variation [21]. Thus, there may be a bias towards a higher phylogenetic to traditional species ratio for molecular studies. Secondly, molecular studies have shown that tropical species tend to show greater levels of differentiation than those of temperate species [22,23], and therefore estimates of the total number of taxa/species worldwide will be biased if the distribution of sampled species does not coincide with the geographic distribution of species diversity. We investigated this by plotting the distribution of phylogroups, generally recognized as reciprocally monophyletic populations on a mtDNA gene tree, as a function of latitude. To explore the relationship between the geographic distribution of biological species diversity, our sampling of biological species and the estimate of phylogenetic diversity, we extracted the average number of biological species in 10-degree latitude blocks from Figure 2 of Blackburn and Gaston [24]. We note there could be other biases, such as greater emphasis on New World taxa. To demonstrate the importance of including phylogroup taxa in evolutionary, ecological and biogeographical studies, we revisited the meta-analysis of Weir and Schluter [25]. They fit birth-death models to plots of molecular divergence dates vs. midpoint latitude, for the current ranges of both biological species (n = 191) and phylogroups (n = 68), and concluded that both diversification and extinction rates are higher at more northerly latitudes (for both categories). To test this conclusion, we used our data set of 260 avian sister lineages (both species and phylogroups) to construct a plot of genetic distance (a proxy for divergence time) vs. the latitude of the midpoint of the current range for comparison.

## Results

### Morphological estimates

**Fig 1:**
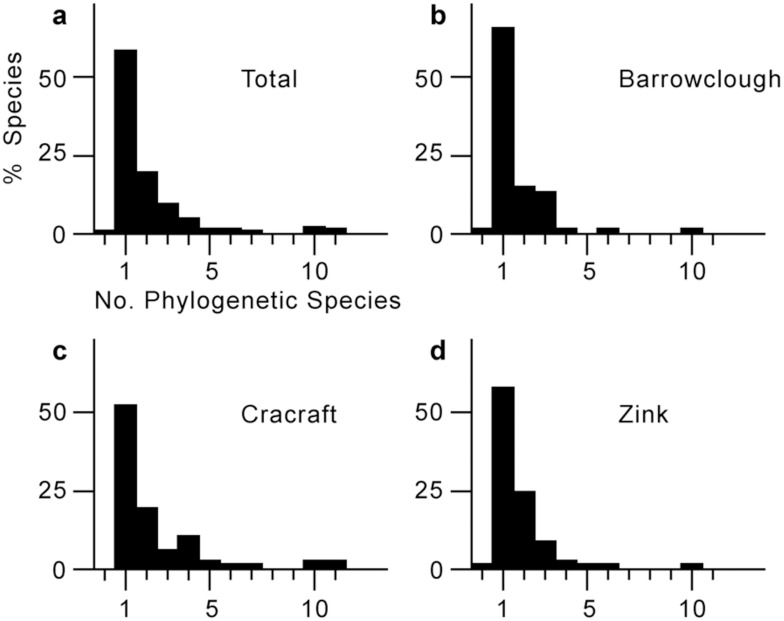
Distribution of numbers of phylogenetic species of birds per biological species. (a) for the total sample of 200, and (b–d) for the individual distributions of three authors.

Estimates of the number of phylogenetic species per biological species ranged from one to 11 (S1 Table). The overall average was 1.97 phylogenetic species per biological species (Fig 1); the distribution for the sample (Fig 1a) is highly skewed with most (59%) biological species containing only a single phylogenetic species, whereas 41% include two or more. Individual distributions of numbers of phylogenetic species produced by the three authors (Fig 1b–1d) all had identical medians (1.0). Means were 1.66, 1.77, and 2.48 for GFB, RMZ, and JC, respectively; these reflect the long tails of the highly non-normal distributions. The three distributions were compared using non-parametric, two-sample Kolmogorov-Smirnov tests; none of the three pairs of distributions differed significantly at the 0.05 level of significance.

Given our sample of 200 exemplars from a universe of 9159 biological species, the ratio of 1.97 can be extrapolated to a total of 18043 phylogenetic species. Because the distribution of the ratios for the sample is non-normal, we used a non-parametric method to investigate confidence limits for this estimate. Bootstrap re-sampling resulted in an empirical 95% confidence interval of 15845 to 20470 for the number of phylogenetic species of extant birds.

Of the 200 species we sampled from the 9159 biological species in Wood and Schnell’s [10] list, subsequent taxonomic changes had occurred in 28 instances in the Howard and Moore list [13,14]; these included 18 splits of single species into two or more species-level taxa, 8 instances of species recognized by Wood and Schnell [10] lumped into other species, and the discovery of two taxonomic errors. Thus, taxonomic adjustments occurring between the two lists resulted in an increase from 200 to 218, or 9%. Adjusting our results for these taxonomic changes, our estimate would be a ratio of 1.76 phylogenetic species per current, eclectic species. For the 10021 such species, this results in an overall estimate of 17606 phylogenetic species, well within the bootstrap confidence interval for the Wood and Schnell [10] list used by the AOU. Consequently, our estimate of a current substantial under-estimation of avian diversity does not depend on the specific classification used in the survey.

**Fig 2:**
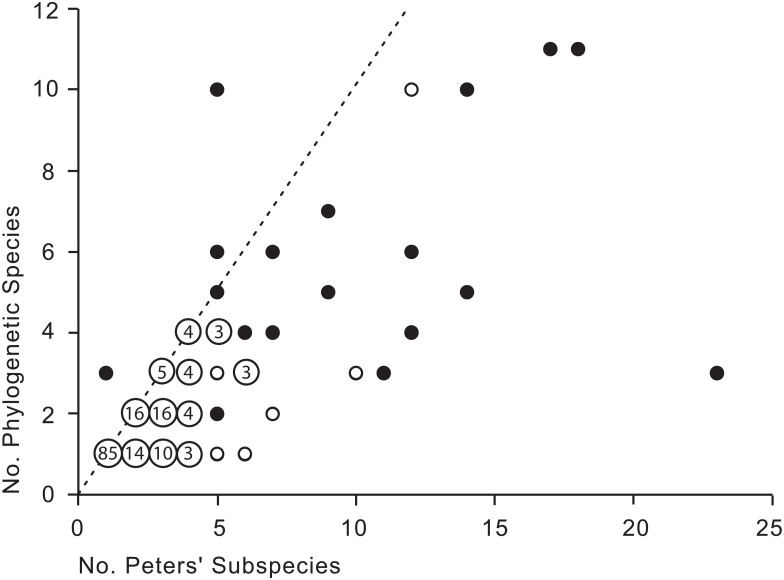
Relationship between the number of generally recognized subspecies (e.g., [8]) within a biological species and the number of phylogenetic species recognized by the authors. The Pearson correlation coefficient is 0.76. The dashed line corresponds to the expectation if all subspecies were equivalent to phylogenetic species. Solid points represent one occurrence, open circles represent two occurrences, and numbers in circles are numbers of occurrences of three or more; complete data are available in S1 Table.

We compared our estimates of phylogenetic species to an estimate of the number of avian subspecies [8]. In this comparison, we excluded two species in our list that are now known to have been described on the basis of aberrant specimens and a third species that was recently described and hence represents a taxon not treated in the appropriate volume of Peters' Check-list. For the remaining 197 species, we found that the number of phylogenetic species and the number of subspecies were significantly (P < 0.0001) correlated (Fig 2). However, in almost all cases our results lie below the line having a slope of one. That is, the estimated number of phylogenetic species is approximately one half of the currently recognized number of subspecies.

### Genetic estimates

**Fig 3:**
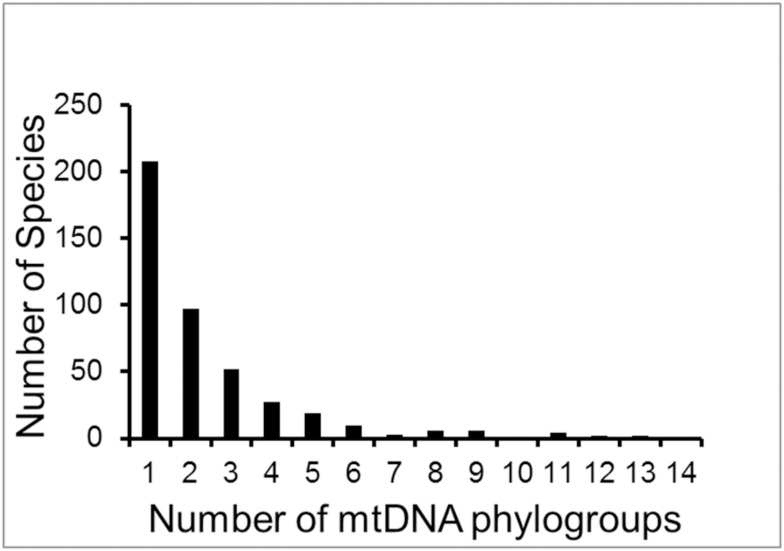
Distribution of distinct taxa per biological species based on mtDNA data. The average is 2.4 evolutionarily significant groups per species.

**Fig 4:**
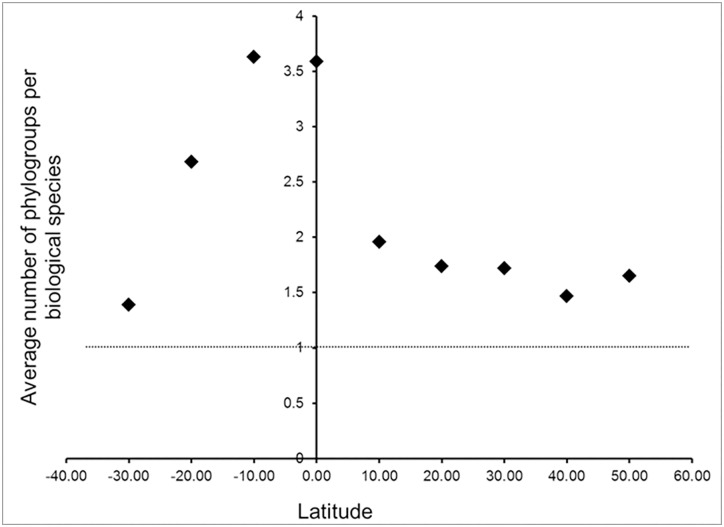
Relationship between geography and species richness. Plot shows average number of phylogroups per biological species as a function of latitude.

**Fig 5:**
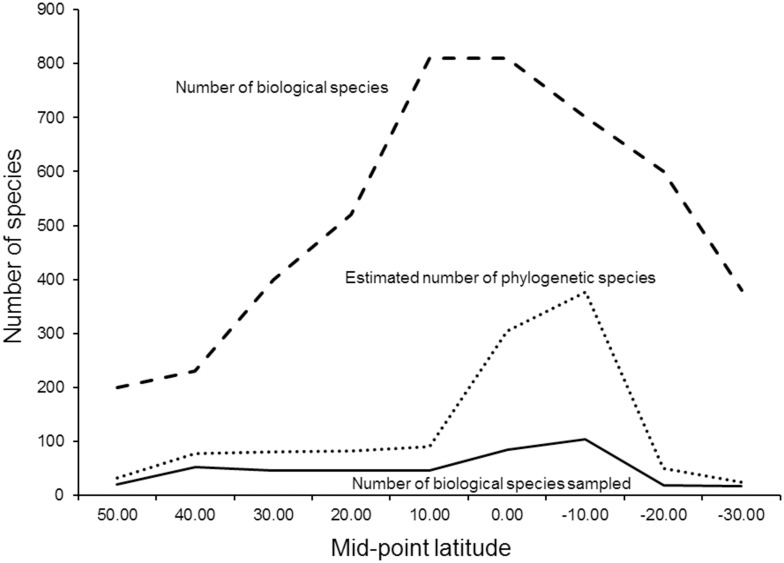
Distribution of species diversity. Number of biological species, number of biological species sampled in this study for mtDNA, and the number of phylogenetic species as a function of latitude.

**Fig 6:**
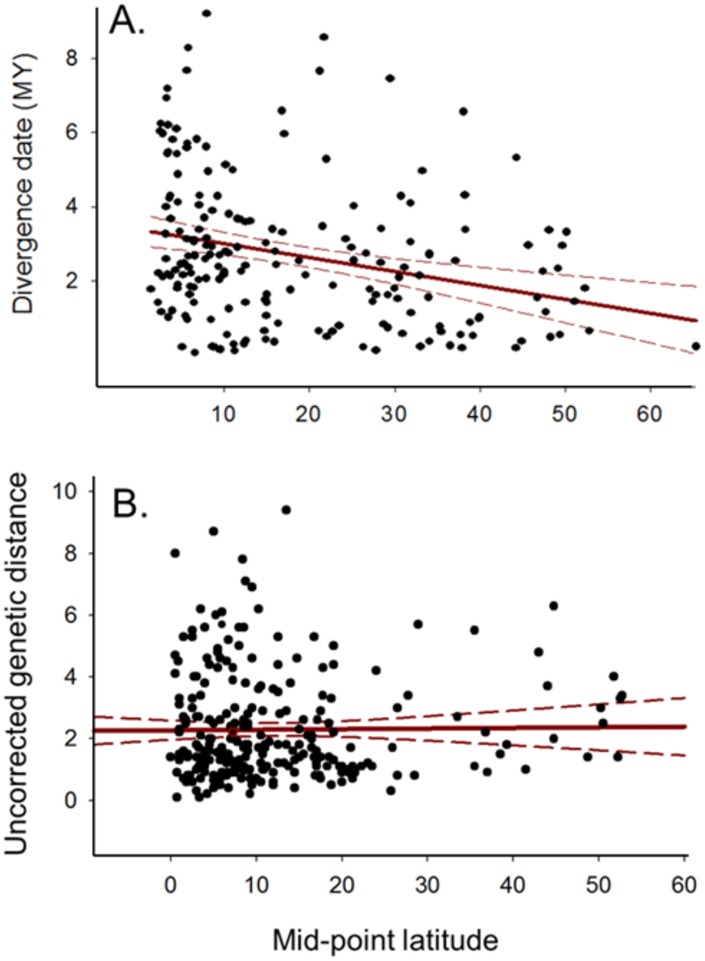
A reconstructed plot [25] for 191 sister species of New World birds suggesting a latitudinal trend in diversification rates. A. Divergence dates were determined using corrected (GTR +) mtDNA data and a 2%/my molecular clock. B. A plot of uncorrected mtDNA genetic distance vs. mid-point latitude for 260 pairs of sister taxa, irrespective of taxonomic rank, suggesting diversification rates do not vary by latitude.

The mtDNA data yielded an estimate of 2.4 taxa per biological species (S2 Table), and the distribution was also left skewed (Fig 3). Although species used in molecular analyses were not randomly sampled, using the same number as for the morphology study (9159) yields an estimate of ca. 22,000 species. Bootstrap re-sampling resulted in an empirical 95% confidence interval of 20,452 to 24,216 for the number of phylogenetic species of extant birds. A plot of the number of phylogroups per biological species versus latitude (Fig 4) showed that for all latitudes, biological species (on average) include more than a single phylogenetic unit. In the New World, we found that the average number of phylogroups per species ranges from 1.4 at 50° N to 3.5 near the equator, with an increasing gradient from high latitudes towards the tropics. Thus, there is not a single average that is appropriate for all geographic regions. Unlike our sampling of species for morphological analyses, the available sample of molecular studies was not random with respect to the geographic distribution of biological species diversity, reflecting some bias in the choices made by molecular systematists in selecting species for study (Fig 5). Our plot of percent sequence divergence vs. midpoint latitude for 260 pairs of sister taxa (Fig 6; S3 Table) revealed no relationship between latitude and rate of diversification (slope = 0.0001).

## Discussion

### The number of bird species

The striking finding of our study is the agreement between morphological and molecular data sets that the number of bird species is approximately twice that recognized under a taxonomy derived from application of the biological species concept, whether the pool of species was more traditional [10] or more recent [13,14]. Because there was little overlap among species considered in the morphological and molecular analyses, these are essentially independent estimates that reveal that the number of taxa of birds relevant for phylogenetic, biogeographic, or conservation analyses is approximately two to 2.5 times the recognized number of biological species. It was not our intent, however, to propose taxonomic revisions for each of the 640 species in the sample. Such an undertaking would require examination of all relevant specimens at many museums and integration of morphological and genetic data for the same species. However, our study provides a glimpse of what a future taxonomy would encompass.

Although our morphological study suggested a world-wide species diversity for birds of 18,043, our molecular study (Fig 3), which we believe is a more comprehensive estimate of taxonomic diversity within biological species, albeit geographically and taxonomically biased across birds, suggests that this number might be an underestimate [26]. This could result from a greater chance of discovering cryptic species with molecular data. Choice of molecular markers could be important in this regard. We do not foresee that mtDNA data will provide a biased estimate of species diversity [27].

We documented a geographic gradient in phylogenetic species diversity from high latitudes towards the tropics. In a multiple regression, both latitudinal midpoint and latitudinal range were significant predictors (P < 0.001) of the number of phylogroups, whereas the areal extent of a species’ range was not significant (P = 0.14). Thus a worldwide estimate based on molecular data should perhaps be weighted by the fact that there are more tropical species and they are relatively more highly subdivided. However, the distribution of species diversity in the tropics also has a significant longitudinal component. For example, at the equator, east-west (biological) species diversity ranges from 418 to 712 per equal sized geographical blocks [28,29]. Thus, determining the precise number of species will require concentrated effort, and we suggest that our estimate likely will be a lower bound.

It has been suggested that the widespread adoption of a phylogenetic species concept by avian systematists would result in an unmanageable number of names [30–32]. A related concern [33] has been the potential variation exhibited by individual systematists when applying the criterion of diagnosability. Our results suggest that these are not major problems. First, 16,000 to 24,000 is not an unmanageably large number of species, given the numbers of species in most other groups of organisms [34]. In addition, a similar ratio of phylogenetic species:biological species has been found in other groups, such as primates [35]. Second, at least given these authors and these samples, there was no significant heterogeneity in recognition of phylogenetic species: the distributions had identical medians, were statistically non-significantly different, and had modest differences in means. The latter reflects differences in the tails of the distributions; these might be due to either differences in the three samples of species or to small differences in interpretation of morphological variation. Taken together, this suggests that, on average, different avian systematists will operationally interpret the phylogenetic species concept more-or-less consistently.

### Consequences of adopting a phylogenetic species concept

Although considerable effort has been expended on establishing the evolutionary history of the earth’s biota, through programs such as the National Science Foundation’s “Genealogy of Life” (https://www.nsf.gov/publications/pub_summ.jsp?ods_key=nsf14527); accessed 15 October 2016), it has been done without a complete understanding of the diversity of units at the twigs of the major branches, namely species. Our analyses show that about one half of the world’s avifauna has been omitted from these efforts, and future studies should determine whether there is an effect on higher-order phylogenetic relationships. In particular, our analyses also suggest that lineage diversity in the tropics is underestimated by a factor of three or more. We anticipate that similar lineage diversity exists in the Old World [36].

### Units of conservation

In the current biodiversity crisis, with many species becoming increasingly threatened, it is important to develop methods for identifying units of conservation and determining how best to allocate conservation resources. Although it has been suggested that the "evolutionary significant units" for conservation and management are phylogenetic species [37], endangered species legislation has codified species, subspecies and distinct population segments (vertebrates only) as the units of conservation for birds in the United States [38] whereas species-level taxa are conservation units in most other countries. It might be argued that for rapid conservation or biogeographic assessment, current subspecies taxonomy might be sufficient for selecting units of conservation. Our results indicate that this is not the case.

Over 17,000 named avian subspecies exist in the taxonomic literature [13,14,30,39], in addition to the nominate taxa. Our morphological analysis yielded an estimate of approximately 18,000 phylogenetic species, or nearly 9,000 phylogenetic species in addition to the approximately 9,100 recognized "biological species;" therefore only about one-half of the generally recognized subspecies are equivalent to phylogenetic taxa (Fig 2). Many described subspecies are insufficiently distinct to be considered either phylogenetic species or "evolutionary significant units" [40]; avian subspecies often include, for example, arbitrary subdivisions of geographic gradients in character variation [41]. This lack of concordance stems in part from the fact that many avian subspecies were described long ago, using few specimens, only morphological characters, and without statistical analyses [42]. It can be argued that their purpose was not to identify units of evolution, but to draw attention to patterns of morphological character variation [43]. Hence, uncritical use of subspecies as proxies for units of conservation can be misleading [44].

Some authors (e.g., [45]) claim that elevation of subspecies to species level constitutes taxonomic inflation and that this could impede conservation [46]. In contrast, Sangster [47] suggested that taxonomic progress, not taxonomic inflation, led to increasing numbers of recognized species of birds. It is not clear that a more accurate understanding of evolutionary diversity is detrimental to conservation practices. In fact, elevation of subspecies to species has often led to increased protection [48]. We argue that taxonomy should reflect patterns of evolutionary taxonomic diversity [43], which becomes clearer when based on a phylogenetic species concept. Hence, we think that a twofold increase in the recognized number of avian species is not taxonomic inflation; instead, it represents a quantum change in the *accuracy* of our understanding of avian diversity. Thus, an evolutionarily faithful classification is best suited to the needs of conservation, and a classification of birds based on the phylogenetic species concept ought to be the framework for conservation.

### Areas of endemism

A phylogenetic classification of the world’s bird species will result in a more refined delineation of the world's areas of endemism [49], which are of critical importance in studies of historical biogeography and diversification [50]. An accurate understanding of areas of endemism is also crucial for identifying and setting priorities on regions for conservation action [51–53]. Such priorities often have been based on surveys of highly visible organisms such as birds, mammals, butterflies and flowering plants, the precise groups in which the use of the biological species concept has been most prevalent [54]. Thorough revisions of these groups will have a substantial impact on empirical studies of evolutionary biology, biogeography, and conservation.

### Inferring patterns of speciation–why taxonomy matters

Species and their geographic distributions, tabulated from regional lists, field guides, or directly from natural history collections, often are used as the basic units of analysis by ecologists and biogeographers (e.g., [24,29]). However, many molecular studies including those surveyed here have shown that currently recognized species often include multiple reciprocally monophyletic units [55]. These units often represent fundamental evolutionary and geographic entities that may not be recognized taxonomically. Unrecognized taxa or biased sampling across geography (Fig 4) could influence comparative studies across a large latitudinal scale. For example, Weir and Schluter’s [25] investigation of latitudinal trends in diversification rates included 191 pairs of New Word avian sister species that were treated as single entities, as well as 68 reciprocally monophyletic units that were included because of “greater taxonomic uncertainty at lower latitudes.” The question becomes whether better accounting of lineage diversity by including lineages “below” the species level (e.g., phylogroups) will influence the conclusion that both diversification and extinction rates (in both data sets) are higher at more northerly latitudes (Fig 6A). Our analysis of 260 lineages (including phylogenetic species) and the resultant plot (Fig 6B) shows no relationship between molecular diversity and latitude. Why results differ between these two studies is unclear but it may reflect the method of measuring taxonomic diversity. Our results show the importance of including all lineages that qualify as independent taxa in comparative studies and not relying on traditional taxonomies for testing evolutionary hypotheses.

## Supporting Information

S1 Appendix Comparison of species concepts. Brief overview of different definitions of species.
(DOCX)

S1 Table The biological species sampled in the morphological study. The number of phylogenetic species estimated in this study, and the number of subspecies recognized by Paynter [8].
(XLSX)

S2 Table Species included in mtDNA comparisons. Number of phylogroups per species as determined by mtDNA sequence data, mid-latitude range estimates, and range sizes. Unless indicated otherwise, taxa occupying small (< 150,000 square miles) and contiguous ranges were assumed to have no genetic structure.
(XLSX)

S3 Table Mitochondrial DNA distances between avian sister taxa. Approximate localities and numbers of individuals used are indicated in parentheses.
(XLSX)
