# Supplementary material for: How Many Kinds of Birds Are There and Why Does It Matter?
Source: PLoS One. 2016 Nov 23;11(11):e0166307. doi: 10.1371/journal.pone.0166307 (PMC5120813; doi:10.1371/journal.pone.0166307)
Supplement: S1 Appendix — Brief overview of different definitions of species. (DOCX) [file pone.0166307.s001.docx]

**S1 Appendix. Comparison of species concepts.** Brief overview of competing definitions of species.

Species play a central role in comparative biology, yet controversies over which entities in nature should be called species have persisted for decades [1-7]. For sexually reproducing organisms, phylogenetic species have been defined as the smallest aggregations of one or more populations that can be distinguished by combinations of heritable characters observed in comparable individuals they are intended to represent the smallest diagnostic lineages in nature with unique evolutionary histories that cannot be further subdivided taxonomically. The biological species concept emphasizes gene flow, reproductive continuity, and the development of reproductive isolating mechanisms [8]; biological species can be composed of multiple evolutionarily independent lineages (e.g. phylogenetic species) but which nonetheless interbreed or are thought to be capable of doing so. In many cases, including sexually dimorphic species or those with multiple developmental stages, both species concepts recognize identical single species.

The fundamental practical difference between the concepts is that diagnosable groups are considered to be species under the phylogenetic species concept, but only so under the biological species concept if they are also reproductively isolated. Thus the ability to interbreed is not evidence of conspecificity under the phylogenetic species concept whereas reproductive isolation is the cornerstone of species status under the biological species concept 7. For example, if in a narrow hybrid zone two distinct populations interbreed, they are often considered conspecific under the biological species concept (even if not each other's sister species), whereas they would constitute separate phylogenetic species, especially if they were not sister species 10,19. Numerous examples exist in birds [9,10].

**References**

1. Ehrlich PR, Raven PH. Differentiation of populations. Science. 1969;165:1228-1232.

2. Sokal RR, Crovello TJ. The biological species concept: a critical evaluation. Am Nat. 1970;104:127-153.

3. McKitrick MC, Zink RM. Species concepts in ornithology. Condor. 1988;90:1-14.

4. De Queiroz K. Species concepts and species delimitation. Syst Biol. 2007;56:879-886.

5. Wheeler QD, Meier R (editors) Species concepts and phylogenetic theory: a debate. New York: Columbia University Press; 2000.

6. Frankham R, Ballou JD, Dudash MR, Eldridge MD, Fenster CB, Lacy RC et al. Implications of different species concepts for conserving biodiversity. Biol Conserv. 2012;153:25-31.

7. Zink RM, McKitrick MC. The debate over species concepts and its implications for ornithology. Auk. 1995;112:701-719.

8. Mayr E. Animal species and evolution. Cambridge, Massachusetts: Belknap Press of Harvard University Press; 1963.

9. Zink RM. Evolution of brown towhees: allozymes, morphometrics and species limits. Condor. 1988;90:72-82.

10. Freeman S, Zink RM. A phylogenetic study of the blackbirds based on variation in mitochondrial DNA restriction sites. Syst Biol. 1995;44:409-420.
